# Rural-urban differences in oral typhoid vaccine responses in Uganda: contribution of *Schistosoma mansoni*

**DOI:** 10.1038/s41541-026-01484-y

**Published:** 2026-05-16

**Authors:** Bridgious Walusimbi, Agnes Natukunda, Rebecca Amongin, Victoria Heyraud, Claire Baine, Jacent Nassuuna, Emily L. Webb, Alison M. Elliott, Gyaviira Nkurunungi

**Affiliations:** 1 Immunomodulation and Vaccines Group, Vaccine Research Theme, Medical Research Council/Uganda Virus Research Institute and London School of Hygiene and Tropical Medicine (MRC/UVRI and LSHTM) Uganda Research Unit, Entebbe, Uganda; 2 Department of Infection Biology, London School of Hygiene and Tropical Medicine, London, UK; 3 International Statistics and Epidemiology Group, Department of Infectious Disease Epidemiology, London School of Hygiene and Tropical Medicine, London, UK; 4 Department of Clinical Research, London School of Hygiene and Tropical Medicine, London, UK; 5 Present Address: School of Computing, Engineering and The Built Environment, Edinburgh Napier University, Edinburgh, UK

**Keywords:** Diseases, Immunology, Medical research, Microbiology

## Abstract

Vaccine immunogenicity varies across populations, yet drivers remain unclear. We compared systemic (plasma) and mucosal (coprological) antibody responses to the oral typhoid vaccine Ty21a, quantified by ELISA, among Ugandan adolescents from urban and *Schistosoma mansoni*-endemic rural settings. Urban participants elicited higher responses. Causal mediation analysis indicated that *S. mansoni* partly accounted for reduced mucosal, but not systemic, responses in rural participants, highlighting implications for oral vaccine effectiveness in endemic settings.

## Introduction

Oral vaccines exhibit heterogeneity in immunogenicity and efficacy across populations, with impaired responses often reported in tropical low-income countries (LICs) compared with high-income countries (HICs)^1,2^, and in rural compared to urban LIC settings^3^. Environmental exposures, including immunomodulators such as helminths, may contribute to this variability, yet their effects remain incompletely understood^4,5^. Using harmonised protocols from the POPulation differences in VACcine responses (POPVAC) programme in Uganda^6,7^, we previously reported rural-urban differences in *Salmonella enterica* serovar Typhi O-lipopolysaccharide (O:LPS)-specific plasma IgG responses following oral typhoid (Ty21a) vaccination^3^, and inverse associations with *Schistosoma mansoni* (*Sm*) infection^8^. Given the importance of mucosal immunity in protection against *S.* Typhi^9^, we extended these findings by evaluating systemic and mucosal antibody responses, focusing on *S*. Typhi O:LPS-specific plasma IgA and copro-IgA and copro-IgG, across contrasting POPVAC settings: *Sm*-endemic rural islands of Lake Victoria^10,11^ and urban mainland communities with lower helminth exposure^12,13^. We used causal mediation analysis to assess whether *Sm* infection contributed to observed rural-urban differences in these responses.

**Fig. 1:**
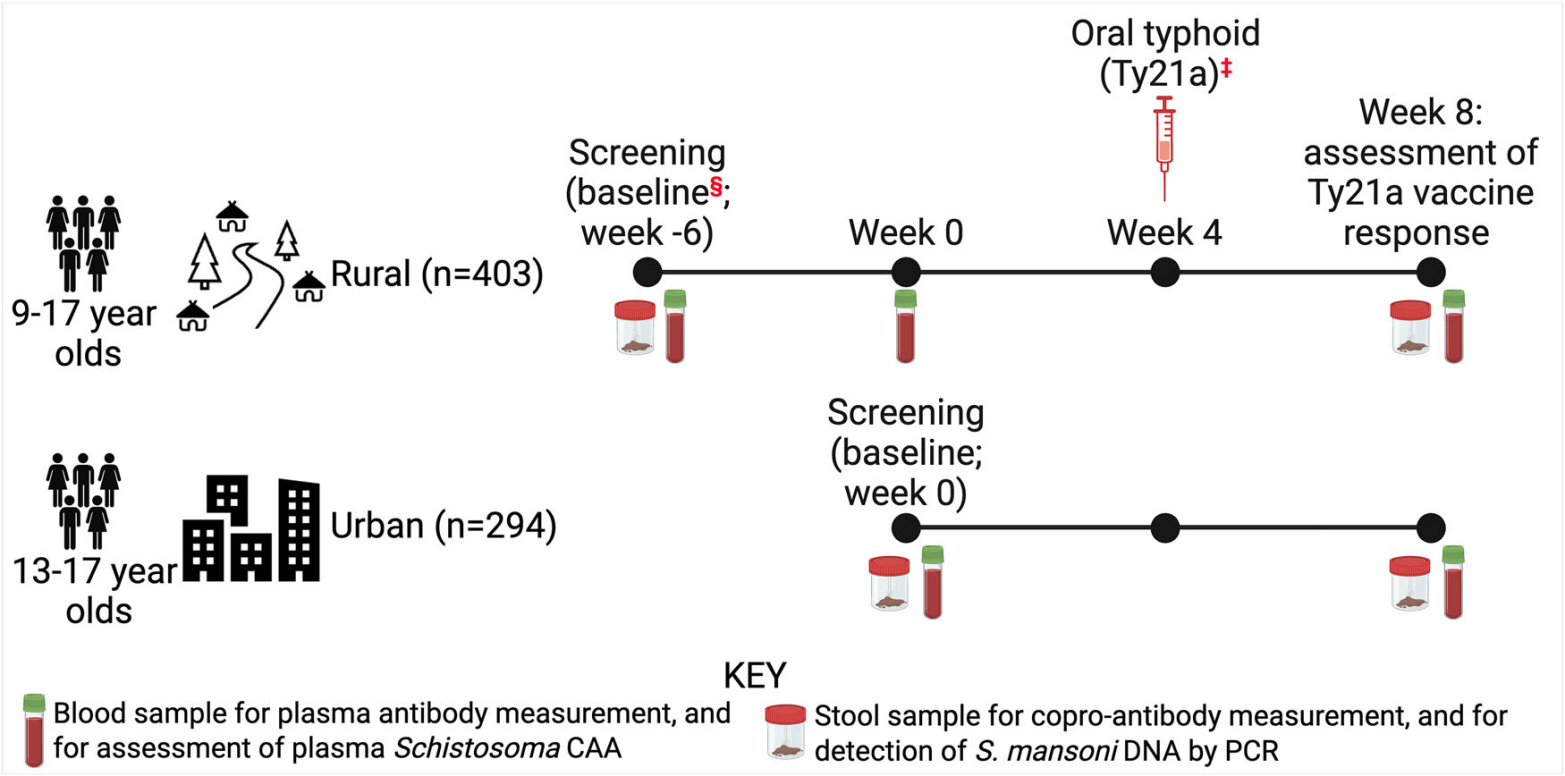
Study schedule. Created in BioRender: https://BioRender.com/6pie25g^§^In the rural trial, baseline *S.* Typhi O:LPS-specific copro-antibodies and total copro-IgA were measured at week-6 (screening timepoint) and baseline *S.* Typhi O:LPS-specific plasma antibodies were measured at week 0. Baseline (screening) for the urban trial was at week 0, as there was no week-6 timepoint. All baseline timepoints were pre-Ty21a vaccination. ^‡^One capsule per day taken on 3 alternate days (day 1, 3 and 5).

Figure 1 and Supplementary Fig. 1a show the study schedule, with additional details provided in the flowchart (Supplementary Fig. 1b) and methods section. Of the 778 participants enrolled across the two POPVAC settings, 718 (92%) were seen at the Ty21a vaccination timepoint. Of these, 699 (97%) received at least one Ty21a dose. Baseline (pre-Ty21a vaccination) data were available for 697 participants (403 rural – POPVAC A; 294 urban – POPVAC C) for at least one of the following measures: *S. Typhi* O:LPS-specific plasma IgA, copro-IgA, copro-IgG, or total copro-IgA (Supplementary Fig. 1b and Table 1). Sex distribution was comparable between settings (57% vs 60% male; *p* = 0.560), but rural participants were younger than urban participants (median age 11 vs 15 years; *p* < 0.001). *Sm* exposure was markedly higher in rural participants, with 71% testing positive by combined plasma circulating anodic antigen (CAA) and/or stool PCR compared with 43% among urban participants (*p* < 0.001). Ty21a uptake was high (≥92% for each of the three doses), but lower among rural participants for doses 2 and 3 (*p* < 0.001; *p* = 0.009, respectively).

**Table 1:** Baseline characteristics of study participants

Characteristics	Rural (POPVAC A)	Urban (POPVAC C)	
	*n*/*N* (%)	*n*/*N* (%)	*p* Value
*Socio-demographic*			
Age in years, median (range)	11 (9–17)	15 (13–17)	<0.001
Male sex	231/403 (57)	175/294 (60)	0.560
*Baseline S. mansoni infection*			
Plasma CAA ≥ 30 pg/ml	218/402 (54)	58/294 (20)	<0.001
PCR positive	237/400 (59)	106/291 (36)	<0.001
PCR positive and/or CAA ≥ 30 pg/ml	286/403 (71)	126/294 (43)	<0.001
*Baseline STH infections*			
*Necator americanus*, PCR positive	87/400 (22)	3/291 (1)	<0.001
*Strongyloides stercoralis*, PCR positive	31/400 (8)	2/291 (1)	<0.001
*Oral typhoid (Ty21a) vaccination*			
Received Ty21a dose 1	403/403 (100)	292/294 (99)	0.097
Received Ty21a dose 2	367/398 (92)	291/294 (99)	<0.001
Received Ty21a dose 3	316/331 (95)	289/292 (99)	0.009

Data are from POPVAC A (rural, *N* = 403) and POPVAC C (urban, *N* = 294) participants who received oral typhoid vaccination and who had baseline data available for at least one of the following measures: total copro-IgA, *Salmonella* Typhi O:LPS-specific copro-IgG, *S*. Typhi O:LPS-specific plasma IgA and *S.* Typhi O:LPS-specific copro-IgA. Denominators vary due to missing data and are therefore lower than 403 (rural) or 294 (urban) for some variables. *P* values for comparisons between rural and urban participants were obtained using chi-squared (or Fisher’s exact) tests for categorical variables and Wilcoxon rank-sum tests for continuous variables.

Abbreviations: *PCR* polymerase chain reaction, *CAA* circulating anodic antigen, *STH* soil-transmitted Helminths.

There was no effect of the POPVAC trial interventions (rural: intensive versus standard praziquantel treatment^10^; urban: BCG versus no BCG revaccination^12^) on antibody concentrations (Supplementary Table 1); therefore, data from both trial arms within each setting were combined for subsequent analyses. Analyses of post-vaccination antibody responses were restricted to participants who received at least one Ty21a dose.

**Fig. 2:**
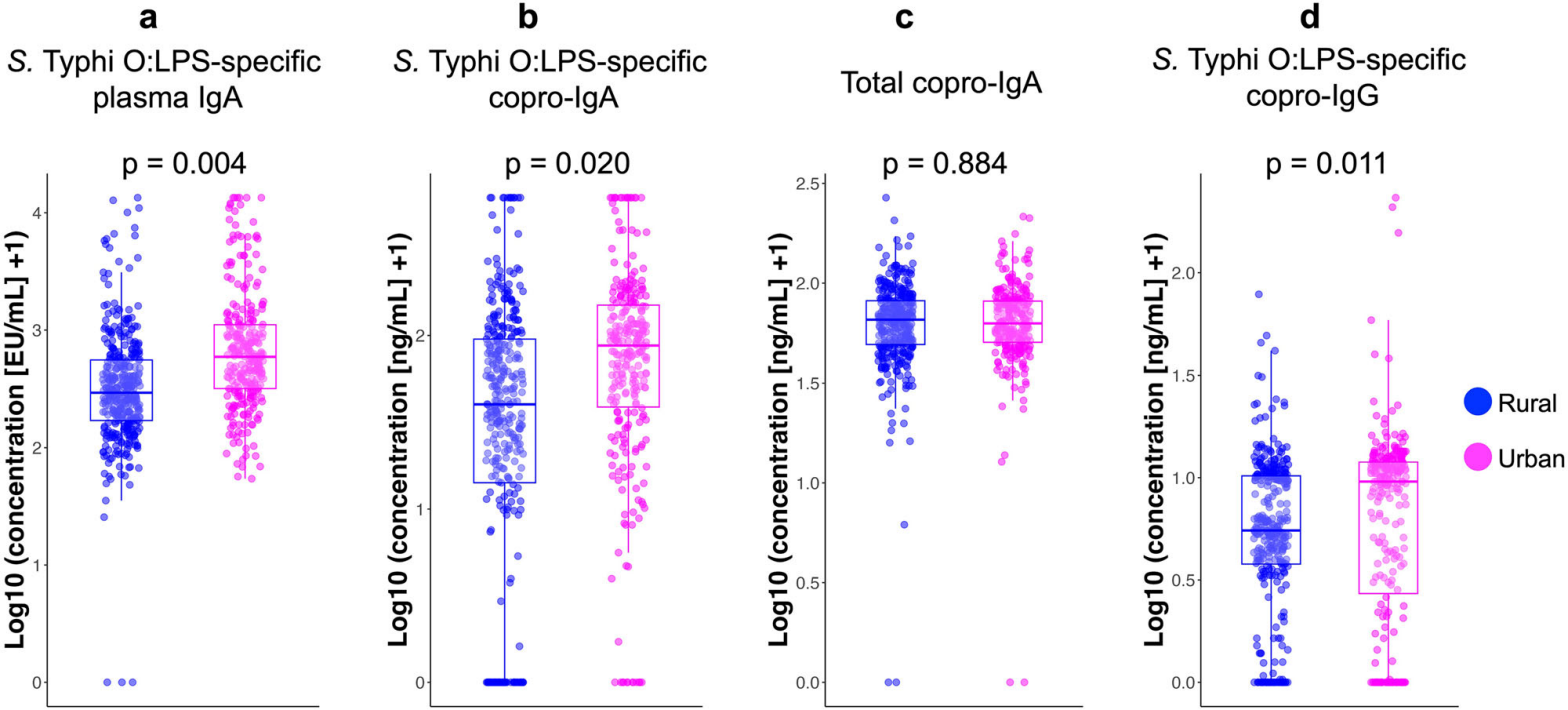
Rural–urban comparison of post-Ty21a vaccination levels of plasma and copro-antibody responses. Linear regression was used to compare (**a**) *Salmonella* Typhi O:LPS-specific plasma IgA, (**b**) *Salmonella* Typhi O:LPS-specific copro-IgA, (**c**) total copro-IgA, and (**d**) *Salmonella* Typhi O:LPS-specific copro-IgG between rural and urban individuals. *P* values are adjusted for age, sex, number of Ty21a doses received and corresponding baseline antibody responses. Horizontal lines in the plots represent medians, and boxes denote interquartile ranges (IQR). Whiskers were drawn using the Tukey method (1.5 times IQR). Individual points represent outliers (>1.5 times IQR away from the median).

Four weeks after Ty21a vaccination, systemic and mucosal *S*. Typhi O:LPS-specific antibody concentrations were substantially higher in urban than rural participants (Fig. 2, Supplementary Table 2): after adjustment for age, sex, number of Ty21a doses received and corresponding baseline antibody levels, urban residence was associated with higher *S*. Typhi O:LPS-specific plasma IgA (geometric mean ratio [95% confidence interval]: 1.44 [1.12-1.84], *p* = 0.004), copro-IgA (1.71 [1.09-2.69], *p* = 0.020) and copro-IgG responses (1.26 [1.05-1.51], *p* = 0.011). By contrast, total copro-IgA levels did not differ significantly by setting, indicating that rural-urban differences reflected antigen-specific vaccine responses rather than global alterations in mucosal IgA production. This likely reflects the composite nature of total copro-IgA, dominated by steady-state mucosal antibody production and continuous environmental antigen exposure. In contrast, antigen-specific vaccine responses require coordinated antigen processing and T cell-dependent class switching, and this might account for greater sensitivity to environmental immunomodulation^14,15^. Consistent with mucosal immunobiology, copro-IgA concentrations were generally higher than copro-IgG, although this observation is interpreted qualitatively owing to assay differences.

**Fig. 3:**
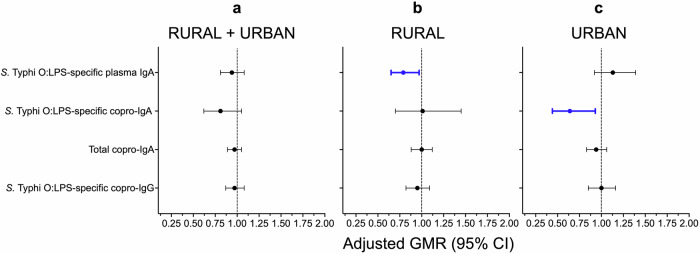
Association of baseline *Schistosoma mansoni* infection with post-vaccination levels of plasma and copro-antibody responses. Forest plots show geometric mean ratios (GMRs) and 95% confidence intervals (95% CIs) for associations between post-vaccination antibody concentrations and current *S. mansoni* infection (plasma circulating anodic antigen ≥ 30 pg/ml and/or PCR-detectable *S. mansoni* DNA) in (**a**) rural and urban participants combined, (**b**) rural participants only, and (**c**) urban participants only. Raw antibody responses were skewed, so log_10_-transformed antibody data were used in our linear regression models; we back-transformed the results to obtain GMRs and 95% CIs. All GMRs and 95% CIs were adjusted for age, sex, setting, number of Ty21a doses received and corresponding baseline responses. Blue colour denotes associations where GMR and 95% CI < 1, and black colour denotes a lack of a significant association. No significant positive associations were observed.

Given the marked disparity in *Sm* exposure between the rural and urban settings, we next assessed associations between baseline *Sm* infection and Ty21a-induced responses using pooled analyses across all participants. In models adjusted for age, sex, geographical setting, number of Ty21a doses and pre-vaccination plasma IgA responses, no significant associations were observed between baseline *Sm* infection and Ty21a-induced responses (Fig. 3a; Supplementary Table 3a). However, interaction tests indicated effect modification by setting for post-vaccination *S*. Typhi O:LPS-specific plasma IgA (interaction *p* = 0.015) and weaker evidence for copro-IgA (interaction *p* = 0.082), suggesting that the immunological impact of *Sm* infection on Ty21a responses differed between rural and urban environments (Supplementary Table 3b).

Stratified analyses supported this interpretation. Among rural participants, *Sm* infection was associated with reduced post-vaccination *S*. Typhi O:LPS-specific plasma IgA (aGMR [95% CI]: 0.79 [0.65-0.97], *p* = 0.021; Fig. 3b, Supplementary Table 3b). This finding is consistent with our previous observations of inverse associations between baseline *Sm* infection and Ty21a-induced plasma IgG in the same rural participants^8^, and with other reports of helminth-associated attenuation of vaccine-specific systemic antibody responses following parenteral immunisation^5,16^. However, *Sm* infection was not associated with post-vaccination copro-IgA responses. In contrast, among urban participants, baseline *Sm* infection was associated with reduced post-vaccination *S*. Typhi O:LPS-specific copro-IgA (0.64 [0.44-0.93], *p* = 0.018), but showed no effect on plasma IgA responses (Fig. 3c, Supplementary Table 3b).

These findings indicate that the immunomodulatory effects of *Sm* infection on oral vaccine-induced immunity are not uniform but are likely contingent on the broader immuno-epidemiological context. In rural settings, sustained exposure to multiple enteric pathogens and chronic immune stimulation may result in a more highly conditioned mucosal immune environment, reducing the marginal impact of helminth infection on vaccine-induced responses^17–19^. High cumulative schistosome exposure in our rural setting may also obscure true inverse associations with Ty21a-induced copro-antibody responses, particularly given evidence that *Sm* infection is associated with increased *Salmonella* intestinal carriage and altered gut ecology^20,21^. Notably, despite this background, we still observed inverse associations between *Sm* infection and plasma Ty21a-induced antibody in rural participants, suggesting that helminth-associated immunomodulation is better detected in the systemic compartment. This may reflect the intravascular lifecycle of *Sm*, with circulating egg antigens driving systemic regulatory immune responses, whereas mucosal effects may be less readily resolved against a background of high local immune activation. In contrast, among urban participants, who experience lower cumulative exposure to helminths and enteric infections, *Sm* infection may exert a more pronounced immunological effect at the intestinal mucosa, resulting in a detectable suppression of antigen-specific mucosal IgA responses following oral vaccination. This interpretation is supported by evidence from our recent work in rural Uganda demonstrating that *Sm* infection is associated with gut inflammation, microbial translocation, and impaired vaccine responses^22^.

Previous studies have shown that *Sm* infection can alter the intestinal microbial community, and mucosal immune function^23–25^, suggesting potential pathways for helminth-mediated modulation of oral vaccine responses. Building on these findings, future studies could evaluate interventions that modulate the gut immune environment, such as dietary strategies, microbiome-directed approaches, or mucosal adjuvants, to enhance oral vaccine immunogenicity in helminth-endemic populations.

To assess whether *Sm* infection may have a causal role in rural-urban differences in Ty21a vaccine responses, we performed causal mediation analyses (Table 2). We observed that rural-urban differences in Ty21a-induced *S. Typhi* O:LPS-specific plasma IgA and copro-IgG responses were largely driven by direct effects of setting, with little evidence of a meaningful contribution from *Sm* infection. However, for *S. Typhi* O:LPS-specific copro-IgA, mediation analysis showed that lower post-vaccination responses in the rural setting were partially mediated by *Sm* infection: while the natural direct effect did not reach statistical significance (GMR [95%CI]: 1.51 [0.91–2.51], *p* = 0.109), the natural indirect effect via *Sm* infection was significant (1.15 [1.02–1.30], *p* = 0.024), corresponding to an estimated 25.52% (95%CI: −6.23 to 57.26) of the total effect mediated. These findings suggest that while rural-urban differences in systemic Ty21a responses are predominantly driven by factors other than *Sm* infection, helminth-associated effects contribute specifically to variation in antigen-specific mucosal IgA responses.

**Table 2:** Estimates comparing schistosomiasis-endemic rural to urban areas mediated by *S. mansoni* infection

Responses, 4 weeks post-vaccination	Total effect GMR (95% CI)	Total effect *p*-value	Natural direct effect GMR (95% CI)	Natural direct effect *p*-value	Natural indirect effect GMR (95% CI)	Natural indirect effect *p*-value	Proportion mediated % (95% CI)
*S. Typhi* O:LPS-specific plasma IgA	1.43 (1.15, 1.76)	0.001	1.48 (1.17, 1.87)	0.001	0.96 (0.90, 1.04)	0.317	-10.35% (-30.80, 10.09)
*S*. Typhi O:LPS-specific copro-IgA	1.74 (1.07, 2.85)	0.026	1.51 (0.91, 2.51)	0.109	1.15 (1.02, 1.30)	0.024	25.52% (-6.23, 57.26)
*S*. Typhi O:LPS-specific copro-IgG	1.26 (1.03, 1.55)	0.027	1.26 (1.02, 1.55)	0.029	1.00 (0.95, 1.06)	0.955	0.66% (-22.21, 23.53)

The table shows the effects of the urban-rural environment on Ty21a responses. Natural indirect effect is the effect of the urban-rural environment mediated through *S. mansoni* infection whereas the natural direct effect is the effect through other pathways. The total effect is the sum of the natural direct and indirect effects. All estimates are adjusted for age, sex, number of Ty21a doses received and corresponding baseline Ty21a responses.

*GMR* geometric mean ratio, *95% CI* 95% confidence interval.

Although *Sm* infection was significantly associated with reduced plasma IgA in rural participants (Supplementary Table 3), a role for *Sm* in explaining the observed rural-urban difference was not supported by mediation analyses, which indicated no detectable indirect effect. The finding that a significant mediation effect was observed only for Ty21a-induced copro-IgA is biologically plausible. Copro-IgA represents a proximal measure of mucosal immune function at the gut-luminal interface, where helminth-associated inflammation, epithelial disruption and microbial translocation are most pronounced. Given that Ty21a is a live attenuated oral vaccine whose immunogenicity relies on effective mucosal antigen processing and IgA class switching, helminth-driven changes of the intestinal immune environment are most likely to manifest as alterations in antigen-specific mucosal IgA responses. Additional environmental exposures, including variation in nutrition, sanitation, enteric infections (including soil-transmitted helminths), microbial diversity and differences in drinking water quality, are likely to shape mucosal immune conditioning beyond the effects of *Sm* infection alone.

Finally, the absence of differences in post-vaccination responses to Ty21a between intensive and standard praziquantel treatment arms suggests that while baseline *Sm* infection contributes to heterogeneity in oral vaccine responses as shown in our mediation analysis, praziquantel treatment alone may be insufficient to reverse established helminth-associated immunomodulation in this high-exposure rural setting, and the treatment–vaccination interval may have been inadequate for such reversal. This likely shows the influence of cumulative environmental exposures, including enteric infections, microbial burden and nutritional factors, that are not addressed by anti-helminthic treatment in isolation.

A potential study limitation is pooling of trial arms; although no overall intervention effects were observed, residual or subgroup-specific effects, including sex-specific responses to intensive praziquantel treatment^10^, cannot be excluded. We also did not formally assess the contribution of soil-transmitted helminths, because they were very rare in the urban setting (Table 1). Furthermore, we did not assess functional antibody activity; therefore, it is unclear whether rural-urban differences in concentrations recapitulate differences in protection. Future studies should incorporate functional, cellular and mechanistic immune profiling.

Together, these data demonstrate pronounced rural-urban differences in immune responses to oral typhoid vaccination, characterised by stronger systemic and mucosal *S. Typhi*-specific antibody responses in urban participants. Our causal mediation analyses suggest that while rural-urban differences in systemic Ty21a responses are predominantly driven by factors other than *Sm* infection, the helminth may contribute to differences in antigen-specific mucosal IgA responses. These findings, based on antibody concentrations and mediation analyses, do not establish causality but highlight environmental exposures as key determinants of heterogeneity in oral vaccine responses.

## Methods

### Ethics statement

Written informed assent was obtained from all participants, and written informed consent from their respective parents or guardians. This included consent for future use of samples for additional related research. Ethics approval was provided by the Uganda Virus Research Institute Research Ethics Committee (references: GC/127/18/09/680, GC/127/19/05/664, GC/127/18/09/682), the London School of Hygiene and Tropical Medicine Observational/Interventions Research Ethics Committee (reference: 16032, 16034), the Uganda National Council for Science and Technology (references: HS2486, HS2491), and the Uganda National Drug Authority (reference: CTA0093, CTA0094).

### Parent study design and population

This analysis uses data and samples from the POPulation differences in VACcine responses (POPVAC) programme, a set of randomised controlled trials^3,26^ among schoolchildren aged 9–17 years in rural^10^ and urban^12^ Uganda.

The rural trial considered here (POPVAC A; July 2019–September 2020; ISRCTN60517191) was conducted in *Schistosoma mansoni-*endemic fishing communities on the Koome islands of Lake Victoria. The full trial protocol^27^ and primary results^10^ have been published. In brief, POPVAC A compared intensive versus standard praziquantel treatment for *Sm* and its impact on vaccine-specific immune responses. Children in the intensive arm received three praziquantel doses (40 mg/kg) before the first vaccination (BCG), followed by quarterly treatment for one year; the standard arm received a single praziquantel dose at the primary endpoint (8 weeks post-BCG), consistent with national school-based deworming guidelines. The POPVAC programme also included a second rural trial (POPVAC B) evaluating intermittent dihydroartemisinin–piperaquine (DP) versus placebo on the same set of vaccine responses^28,29^. As the present investigation did not use POPVAC B samples, all references to the “rural setting” relate exclusively to POPVAC A.

The urban trial (POPVAC C; August 2020–August 2021; ISRCTN10482904)^12,30^ was undertaken in Entebbe municipality, an area with lower helminth and malaria prevalence. POPVAC C evaluated the effect of BCG revaccination compared with no revaccination on immune responses to subsequently administered unrelated vaccines among participants of the Entebbe Mother and Baby Study (EMaBS) cohort^31^. POPVAC C served as the urban comparator for both the wider POPVAC programme^3^ and for this investigation.

Except for the respective trial interventions (praziquantel in POPVAC A and BCG revaccination in POPVAC C), study procedures were harmonised across sites. In both settings, participants received BCG vaccine (Serum Institute of India) at week 0; yellow fever (YF-17D, Sanofi Pasteur, France), oral typhoid (Ty21a, Vivotif, PaxVax, UK; one capsule per day taken on 3 alternate days) and HPV (Gardasil, Merck & Co, USA) at week 4.

### Current study design and population

The present investigation used samples from randomly selected POPVAC A (rural) and POPVAC C (urban) participants and focuses on the Ty21a vaccine. Vaccine immunogenicity (*S*. Typhi O:LPS-specific plasma IgA; *S*. Typhi O:LPS-specific copro-IgG and IgA; procedures detailed below) was assessed at four weeks post-Ty21a vaccination (week 8 of the trials). Total copro-IgA was also measured. Baseline responses were measured at week 0 in the urban trial. In the rural trial, baseline *S*. Typhi O:LPS-specific plasma antibodies were measured at week 0 and baseline total copro-IgA and *S*. Typhi O:LPS-specific copro-antibodies at week -6 (Supplementary Fig. 1), reflecting pre-vaccination sample availability within the parent trial design.

*Schistosoma mansoni* infection status was assessed retrospectively at baseline, prior to any intervention (week-6 in the rural setting and week 0 in the urban setting), using previously published methods^8,10^. Infection was defined by the presence of detectable plasma circulating anodic antigen (CAA), measured using the up-converting phosphor lateral flow SCAA20 assay with a positivity threshold of 30 pg/ml^32^, and/or detection of *S. mansoni* DNA in stool by PCR, adapted from established protocols^33^. Infection with other *Schistosoma* species has not been documented in our study areas^34^. The stool PCR assay also included primers and probes for *Necator americanus* and *Strongyloides stercoralis* DNA detection, as previously reported^10^.

### ELISA measurement of *Salmonella* Typhi O-lipopolysaccharide (O:LPS)-specific copro-IgG and IgA

*Salmonella* Typhi O:LPS-specific copro-IgG and IgA were quantified using in-house ELISAs. Firstly, stool samples (0.125 g) were suspended in 1 ml of 1× phosphate-buffered saline (PBS), vortexed for 1 min and centrifuged at 3000 g for 10 minutes. Supernatants were stored at −80 °C until analysis. On the first day of the ELISA assay, Immulon 4HX 96-well plates (Thermo Fisher) were coated overnight at 4 °C with 50 µL/well of S. Typhi O:LPS (Sigma L2387) diluted in bicarbonate buffer (0.1 M Na_2_CO_3_/NaHCO_3_, pH 9.6), excluding the first two columns. Plates were coated at 15 µg/ml for IgA assays and 5 µg/ml for IgG assays. Twofold serial dilutions of IgA or IgG standards, prepared in coating buffer, were added to the first two columns to generate standard curves. Human colostrum IgA (Sigma I2636; top concentration 500 ng/ml) and human serum IgG (Sigma I2511; top concentration 2000 ng/ml) were used as standards. After coating, plates were washed with 0.05% Tween-20 in PBS (PBST) and blocked for 1 hour at room temperature with 5% skimmed milk powder in PBST. Plates were then washed, and 50 µL of stool supernatants were added and incubated for 2 h at room temperature. For the standard curve wells (first two columns), 50 µL of 1× PBS was added in place of stool supernatant. Following washing, plates were incubated for 1 h with 50 µL/well of either goat anti-human IgA-HRP (Bio-Rad, STAR141P; 1:6000 dilution) or polyclonal rabbit anti-human IgG-HRP (Agilent Dako; 1:3000 dilution), diluted in assay buffer (1% skimmed milk powder in PBST). After a final wash, plates were developed in the dark with 100 µL/well of 3,3′,5,5′-tetramethylbenzidine (TMB; Sigma-Aldrich, T0440) for IgA assays or o-phenylenediamine (OPD; Sigma-Aldrich, P9187) for IgG assays. Colour development was stopped with 30 µL/well of 2 M sulphuric acid after 12 min (IgA) or 17 min (IgG). Optical density was read at 450 nm for IgA assays and 490 nm for IgG assays (reference wavelength 630 nm) using a BioTek ELx808 plate reader. Copro-antibody concentrations (ng/ml) were interpolated from standard curves using Gen5 software (BioTek Instruments Inc., USA) with a five-parameter logistic curve fit.

### ELISA measurement of total copro-IgA

Stool samples were processed as described above. Immulon 4HX 96-well plates (Thermo Fisher) were coated overnight at 4 °C with 50 µl/well of purified goat anti-human immunoglobulins (polyvalent; 1 µg/mL; Invitrogen) diluted in carbonate/bicarbonate coating buffer (0.1 M Na_2_CO_3_/NaHCO_3_, pH 9.6). Plates were washed with 1× phosphate-buffered saline containing 0.05% Tween-20 (PBST) and blocked for 2 h at room temperature with 5% skimmed milk in PBST. After washing, 50 µl/well of standards or samples were added. Standards (human colostrum IgA; Sigma I2636) were prepared in assay buffer (1% skimmed milk in PBST), starting at 500 ng/mL with two-fold serial dilutions, and added to columns 1–2. Stool supernatants were added to columns 3–12, and plates were incubated for 2 hours at room temperature. Plates were then washed and incubated for 1 h at room temperature with 50 µl/well of goat anti-human IgA conjugated to horseradish peroxidase (HRP; Bio-Rad, STAR141P) diluted 1:6000 in assay buffer. After additional washes, plates were developed in the dark with 100 µl/well of o-phenylenediamine substrate (Sigma-Aldrich), and colour development was stopped by addition of 30 µl/well of 2 M sulphuric acid. Optical density was read at 490 nm with a reference wavelength of 630 nm using a BioTek ELx808 microplate reader. Total copro-IgA concentrations (ng/mL) were interpolated from standard curves using Gen5 data collection and analysis software (BioTek Instruments Inc., USA) with a five-parameter logistic curve fit.

### ELISA measurement of plasma Salmonella Typhi O-lipopolysaccharide (O:LPS)-specific IgA

Plasma IgA specific to *Salmonella* Typhi O-lipopolysaccharide (O:LPS) was also quantified using an in-house ELISA. Nunc Maxisorp 96-well plates (Thermo Fisher) were coated overnight at 4 °C with 50 µl/well of *S*. Typhi O:LPS (Sigma L2387) diluted in bicarbonate buffer (0.1 M Na_2_CO_3_/NaHCO_3_, pH 9.6). Plates were coated at 5 µg/ml. Plates were washed with 0.05% Tween-20 in 1× phosphate-buffered saline (PBST) and blocked for 1 h at room temperature with 5% skimmed milk powder in PBST. After washing, plates were incubated for 2 h at room temperature with 50 µl/well of diluted plasma samples and twofold serial dilutions of standard sera. Plasma samples were diluted 1:83.3 in assay buffer (1% skimmed milk in PBST). Standards were derived from pooled sera with known O:LPS-specific antibody titres (top concentration 20 ELISA units [EU]/ml), provided by the Oxford Vaccine Centre Biobank. These sera were obtained from individuals with high O-antigen responses following *S*. Typhi challenge in a controlled human infection study^35^. Following sample incubation, plates were washed, and antibody binding detected by incubation for 1 hour at room temperature with goat anti-human IgG-HRP (Insight Biotechnology, UK) diluted 1:6000 in assay buffer. Plates were washed and developed with 100 µl/well of o-phenylenediamine (OPD; Sigma-Aldrich). Colour development was stopped with 30 µl/well of 2 M sulphuric acid after 10 min. Optical density was measured at 490 nm with a reference wavelength of 630 nm using a BioTek ELx808 plate reader. O:LPS-specific plasma IgA titres were expressed as nominal ELISA units (EU/mL) and interpolated from standard curves using Gen5 data collection and analysis software (BioTek Instruments Inc., USA) with a five-parameter logistic curve fit.

### Statistical analysis

Statistical analyses were conducted in Stata v19.5 (College Station, Texas, USA), with data visualisation done in GraphPad Prism v10.0.0 (GraphPad Software, San Diego, CA, USA). All analyses were conducted only in study participants who were vaccinated with the oral typhoid (Ty21a) vaccine, excluding those who received none of the three Ty21a vaccine doses. Baseline characteristics of study participants were summarised by geographical setting. *Schistosoma mansoni* prevalence and intensity at baseline (week-6, pre-Ty21a vaccination), Ty21a uptake and sociodemographic characteristics were compared between rural (POPVAC A) and urban (POPVAC C) settings using chi-squared (or Fisher’s exact) tests (categorical variables) and Wilcoxon rank sum tests (continuous variables). Analysis of total copro-IgA and *Salmonella* Typhi O:LPS-specific plasma and copro-antibody concentrations revealed skewed distributions; therefore, log_10_ transformations (concentration + 1) were applied to the data. The primary analysis outcomes were *Salmonella* Typhi O:LPS-specific plasma and copro-antibody concentrations, and total copro-IgA, measured 4 weeks post-Ty21a vaccination, at the week 8 timepoint of the POPVAC trials.

For rural-urban comparisons of log_10_-transformed plasma and copro-antibody concentrations, linear regression analyses were conducted. Regression coefficients were back-transformed to obtain geometric mean ratios (GMRs) and 95% confidence intervals (95% CIs). Both crude (unadjusted) and adjusted GMRs and 95% CIs are presented. Adjusted analyses controlled for age, sex, number of Ty21a doses received, and baseline (pre-vaccination) plasma and copro-antibody concentrations.

Associations between baseline *Schistosoma mansoni* infection and post-vaccination plasma and copro-antibody concentrations were also assessed using linear regression. In the primary model, we adjusted for age, sex, number of Ty21a doses received, geographical setting and baseline (pre-vaccination) plasma and copro-antibody concentrations. In the second model, we included an interaction term between *S. mansoni* infection and geographical setting, and used this model to obtain stratum (setting)-specific estimates.

To evaluate whether *Schistosoma mansoni* infection mediated rural-urban differences in Ty21a-induced immune responses, we performed causal mediation analyses within the counterfactual framework using the Stata “mediate” command. Geographical setting (rural vs urban) was the exposure, baseline *S. mansoni* infection (CAA and/or stool PCR positivity) the mediator and post-vaccination antigen-specific plasma and copro-antibody concentrations the continuous outcomes. Analyses were adjusted for age, sex, number of Ty21a doses received and baseline antibody concentrations, and natural direct, indirect and total effects were estimated, with the proportion mediated reported when the effects were in the same direction. This approach assumes no uncontrolled confounding of the exposure-outcome, mediator-outcome, or exposure-mediator relationships. Participants with missing vaccine or mediator data were excluded.

The effects on post-vaccination plasma and copro-antibody concentrations of intensive versus standard praziquantel treatment (rural trial) and of BCG versus no BCG revaccination (urban trial) were also assessed using linear regression. These analyses did not adjust for covariates, as the POPVAC trials were randomised. However, we controlled for corresponding baseline vaccine responses to increase the precision of analyses.

A 5% significance level was used for all analyses.

## Supplementary information

5. Supplementary information_copro_CLEAN COPY

## Data Availability

The de-identified individual participant data that underlie the results reported in this article are stored in a non-publicly available repository (LSHTM Data Compass), together with a data dictionary. Data are available on request via 10.17037/DATA.00005144. Researchers who would like to access the data may submit a request through LSHTM Data Compass, detailing the data requested, the intended use for the data, and evidence of relevant experience and other information to support the request. The request will be reviewed by the corresponding authors in consultation with the MRC/UVRI and LSHTM data management committee, with oversight from the UVRI and LSHTM ethics committees. In line with the MRC policy on Data Sharing, there will have to be a good reason for turning down a request. Patient Information Sheets and consent forms specifically referenced making anonymised data available, and this has been approved by the relevant ethics committees. Researchers given access to the data will sign data sharing agreements, which will restrict the use to answering pre-specified research questions.
