# Supplementary material for: Rural-urban differences in oral typhoid vaccine responses in Uganda: contribution of Schistosoma mansoni
Source: NPJ Vaccines. 2026 May 16;11:155. doi: 10.1038/s41541-026-01484-y (PMC13439054; doi:10.1038/s41541-026-01484-y)
Supplement: Supplementary file 1 [file 41541_2026_1484_MOESM1_ESM.pdf]

SUPPLEMENTARY INFORMATION FOR

Rural-urban differences in oral typhoid vaccine responses in Uganda: contribution of *Schistosoma mansoni*

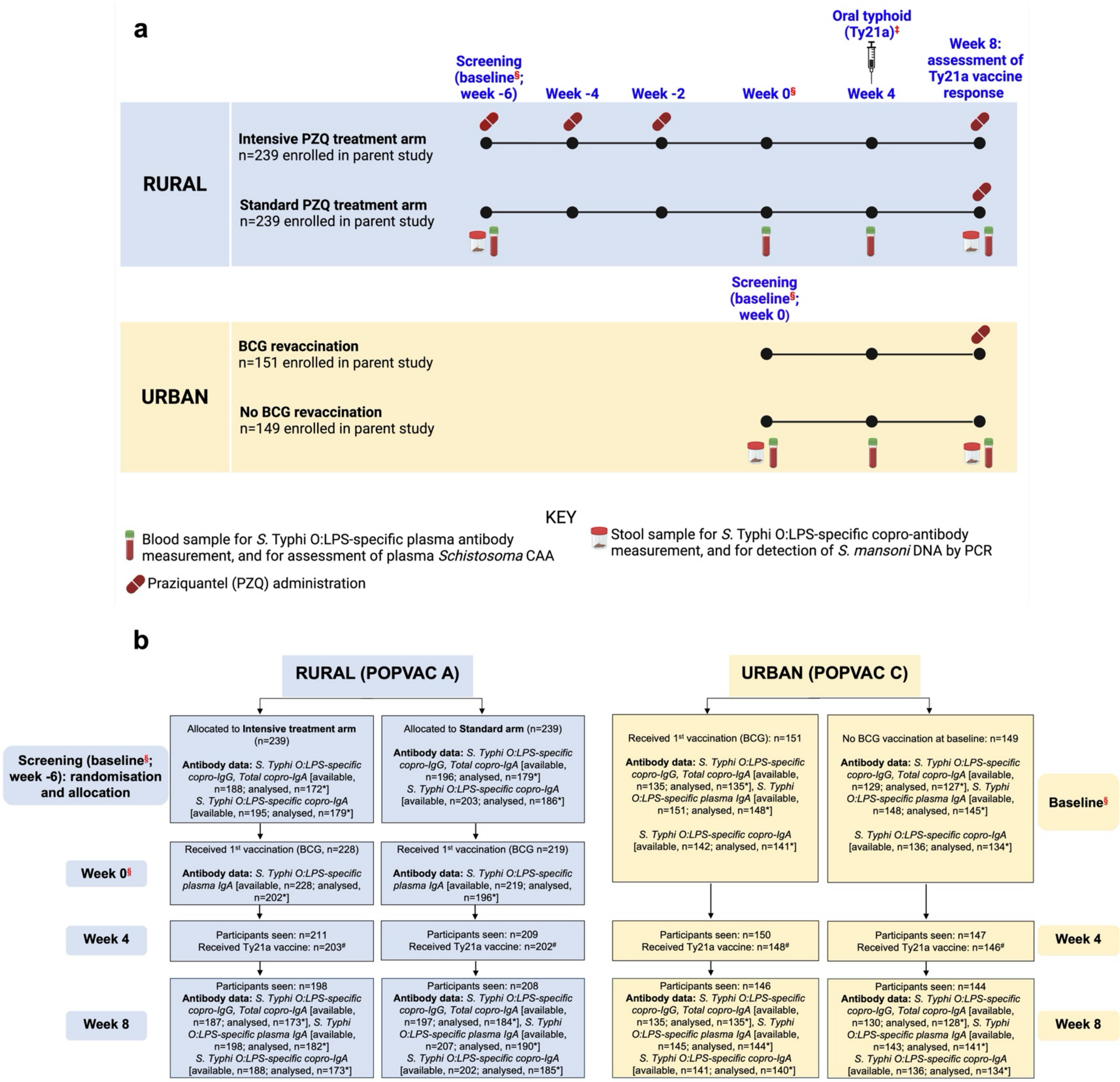

Supplementary Figure 1. Study design (a) Study schedule. Created in BioRender: https://BioRender.com/80cssi4. **(b)** Flow diagram.

§In the rural trial, baseline *S.* Typhi O:LPS-specific copro-antibodies and total copro-IgA were measured at week -6 (screening timepoint) and baseline *S.* Typhi O:LPS-specific plasma antibodies were measured at week 0. Baseline (screening) for the urban trial was at week 0, as there was no week -6 timepoint. All baseline timepoints were pre-Ty21a vaccination.

‡Ty21a (one capsule (dose) per day taken on 3 alternate days, i.e. days 1, 3 and 5) was co-administered with the yellow fever and HPV vaccines

*Statistical analysis conducted only for those vaccinated with at least one of three oral typhoid vaccine (Ty21a) doses at week 4

#Received at least one of the three Ty21a vaccine doses

Supplementary Table 1: Impact of POPVAC trial interventions on post-vaccination antibody levels

Rural (POPVAC A)				
Responses, 4 weeks post-vaccination	Geometric mean (µg/ml)		GMR (95%CI)	p-value
	Standard praziquantel treatment*	Intensive praziquantel treatment		
S. Typhi O:LPS-specific plasma IgA (EU/mL)	307.96	329.33	1.12 (0.95, 1.32)	0.172
S. Typhi O:LPS-specific copro-IgA (ng/mL)	29.81	25.15	0.79 (0.55, 1.12)	0.185
Total copro-IgA (ng/mL)	63.62	59.62	0.94 (0.85, 1.03)	0.195
S. Typhi O:LPS-specific copro-IgG (ng/mL)	5.50	5.03	0.89 (0.80, 1.01)	0.060
Urban (POPVAC C)				
	No BCG revaccination*	BCG revaccination		
S. Typhi O:LPS-specific plasma IgA (EU/mL)	683.83	644.36	0.96 (0.77, 1.19)	0.689
S. Typhi O:LPS-specific copro-IgA (ng/mL)	61.81	70.72	1.14 (0.82, 1.57)	0.435
Total copro-IgA (ng/mL)	60.41	63.97	1.04 (0.91, 1.18)	0.582
S. Typhi O:LPS-specific copro-IgG (ng/mL)	5.95	6.02	0.97 (0.82,1.15)	0.732

Abbreviations: GMR, geometric mean ratio; 95% CI, 95% confidence interval
*Reference category
‡Adjusted for corresponding baseline antibody responses

Supplementary Table 2. Rural-urban comparison of post-Ty21a vaccination levels of plasma and copro-antibody responses, adjusted for baseline responses.

Responses, 4 weeks post-vaccination	Geometric mean		cGMR (95% CI)	p	aGMR (95% CI)‡	p
	*Rural**	*Urban*				
S. Typhi O:LPS-specific plasma IgA (EU/mL)	318.23	663.60	**2.09 (1.75, 2.49)**	**<0.001**	**1.44 (1.12, 1.84)**	**0.004**
S. Typhi O:LPS-specific copro-IgA (ng/mL)	27.45	66.21	**2.41 (1.86, 3.12)**	**<0.001**	**1.71 (1.09, 2.69)**	**0.020**
Total copro-IgA (ng/mL)	61.63	62.21	1.01 (0.93, 1.10)	0.828	1.01 (0.87, 1.17)	0.884
S. Typhi O:LPS-specific copro-IgG (ng/mL)	5.25	5.98	1.14 (0.98, 1.33)	0.101	**1.26 (1.05, 1.51)**	**0.011**

Significant associations (p<0.05) are highlighted in bold.
Abbreviations: cGMR, crude geometric mean ratio; aGMR, adjusted geometric mean ratio; 95% CI, 95% confidence interval
*Reference category is rural setting
‡Adjusted for corresponding baseline antibody responses, age, sex, and number of Ty21a doses received

Supplementary Table 3. Effect of baseline *S. mansoni* infection on pre- and post-vaccination levels of plasma and copro-antibody responses

a. OVERALL (RURAL + URBAN)						
Responses, 4 weeks post-vaccination	Geometric mean		cGMR (95%CI)	p	aGMR (95%CI)‡	p
	*Uninfected**	*S. mansoni infected*				
***S.* Typhi O:LPS-specific plasma IgA (EU/mL)**	510.77	393.03	**0.77 (0.64, 0.93)**	**0.006**	0.94 (0.81, 1.08)	0.389
***S.* Typhi O:LPS-specific copro-IgA (ng/mL)**	51.47	33.97	**0.66 (0.50, 0.86)**	**0.003**	0.81 (0.62, 1.05)	0.109
**Total copro-IgA (ng/mL)**	63.43	60.82	0.96 (0.88, 1.04)	0.330	0.97 (0.89, 1.05)	0.439
***S.* Typhi O:LPS-specific copro-IgG (ng/mL)**	5.76	5.41	0.94 (0.80, 1.10)	0.433	0.97 (0.87, 1.08)	0.552

b. SETTING-SPECIFIC ESTIMATES								
Responses, 4 weeks post-vaccination		Geometric mean		cGMR (95%CI)	p	aGMR (95% CI)‡	p	Interaction p†
		*Uninfected**	*S. mansoni infected*					
***S. Typhi* O:LPS-specific plasma IgA (EU/mL)**	Rural	365.74	300.63	0.82 (0.64, 1.06)	0.128	**0.79 (0.65, 0.97)**	**0.021**	**0.015**
	Urban	638.15	698.65	1.10 (0.83, 1.44)	0.514	1.13 (0.92, 1.39)	0.246	
***S.* Typhi O:LPS-specific copro-IgA (ng/mL)**	Rural	25.31	28.36	1.12 (0.74, 1.71)	0.594	1.01 (0.70, 1.45)	0.960	0.082
	Urban	82.12	50.02	**0.61 (0.44, 0.85)**	**0.003**	**0.64 (0.44, 0.93)**	**0.018**	
**Total copro-IgA (ng/mL)**	Rural	61.68	61.62	1.00 (0.89, 1.13)	0.986	1.00 (0.88, 1.12)	0.922	0.497
	Urban	64.63	59.08	0.91 (0.80, 1.04)	0.177	0.94 (0.83, 1.06)	0.305	
***S.* Typhi O:LPS-specific copro-IgG (ng/mL)**	Rural	6.15	4.92	**0.80 (0.66, 0.98)**	**0.030**	0.95 (0.82, 1.09)	0.448	0.634
	Urban	5.51	6.68	1.21 (0.93, 1.58)	0.157	1.00 (0.85, 1.16)	0.951	

Significant associations (p≤0.05) are highlighted in bold.
Abbreviations: cGMR, crude geometric mean ratio; aGMR, adjusted geometric mean ratio; 95% CI, 95% confidence interval
*Reference category is uninfected group
‡Adjusted for age, sex, setting, number of Ty21a doses received and baseline vaccine responses
†Interaction between *S. mansoni* infection status and setting
